# Complex Pignistic Transformation-Based Evidential Distance for Multisource Information Fusion of Medical Diagnosis in the IoT

**DOI:** 10.3390/s21030840

**Published:** 2021-01-27

**Authors:** Fuyuan Xiao

**Affiliations:** School of Computer Science and Engineering, University of Electronic Science and Technology of China, Chengdu 611731, China; complexbpa@163.com

**Keywords:** complex evidence theory, Dempster–Shafer evidence theory, distance measure, complex mass function, complex pignistic transformation, betting commitment, multisource information fusion, multi-attribute decision-making, medical diagnosis, Internet of Things (IoT)

## Abstract

Multisource information fusion has received much attention in the past few decades, especially for the smart Internet of Things (IoT). Because of the impacts of devices, the external environment, and communication problems, the collected information may be uncertain, imprecise, or even conflicting. How to handle such kinds of uncertainty is still an open issue. Complex evidence theory (CET) is effective at disposing of uncertainty problems in the multisource information fusion of the IoT. In CET, however, how to measure the distance among complex basis belief assignments (CBBAs) to manage conflict is still an open issue, which is a benefit for improving the performance in the fusion process of the IoT. In this paper, therefore, a complex Pignistic transformation function is first proposed to transform the complex mass function; then, a generalized betting commitment-based distance (BCD) is proposed to measure the difference among CBBAs in CET. The proposed BCD is a generalized model to offer more capacity for measuring the difference among CBBAs. Additionally, other properties of the BCD are analyzed, including the non-negativeness, nondegeneracy, symmetry, and triangle inequality. Besides, a basis algorithm and its weighted extension for multi-attribute decision-making are designed based on the newly defined BCD. Finally, these decision-making algorithms are applied to cope with the medical diagnosis problem under the smart IoT environment to reveal their effectiveness.

## 1. Introduction

The Internet of Things (IoT) refers to a very large network, which connects various devices for intelligent identification, locating, tracking, monitoring, and management [1,2]. Thanks to the development of information and communication technologies, the IoT is becoming ubiquitous in all kinds of applications. On the other hand, it is well known that clinical medical service is complex, in which the collection, preservation, analysis, and fusion of patient information takes much time and labor many materials [3,4]. Furthermore, traditional medical systems cannot diagnose patients with a high level decision-making due to a single information source, and this affects the quality of clinical medical service. Therefore, the emergence of the IoT becomes a milestone in the field of the digital medical domain. Medical diagnosis under the smart IoT environment can send physiological information and medical signals through communication networks to monitoring systems for analyzing and diagnosing with the aid of artificial intelligence techniques [5,6,7]. Consequently, it is beneficial for improving clinical medical services and lowering management costs, so that it can help to make better lifestyle and disease prevention plans and personalized medical services for patients [8,9]. In particular, in the data processing of the medical IoT, data fusion technology under uncertain environment plays a very important role, which is effective for processing mass data from multiple sources to better support medical decision-making. Hence, in this paper, we focus on improving the performance of the fusion process under an uncertain medical IoT.

As is well known, Dempster–Shafer evidence theory (DSET) [10,11] has several desirable characteristics to deal with the uncertainty problem. To be specific, the mass function (MF), also called basis belief assignment (BBA), in DSET can express uncertainty quantitatively [12]. Additionally, the Dempster rule of combination (DRC) in DSET can fuse multisource information to reduce uncertainty in the fusion process for supporting decision-making well [13,14,15]. Meanwhile, the DRC meets commutative and associative laws [16,17]. Hence, DSET has been extensively researched, including the aspects of D numbers [18,19], evidential reasoning [20], heuristic representation learning [21], entropy [22,23], generation [24,25], dependency [26], the negation [27] of BBAs, etc. [12]. In particular, DSET was recently well exploited by Xiao [28,29] for the complex plane for handling more complex uncertainty problems, called complex evidence theory (CET).

In CET [28,29], the classical MF is extended to the complex mass function, also called complex basis belief assignment (CBBA), to express uncertainty quantitatively, in which the complex mass function is expressed by complex numbers, not just positive real numbers. In addition, the classical DRC is generalized to fuse CBBAs to better support decision-making, which also satisfies commutative and associative laws. Therefore, since CET is complex-value modeled with the aid of the two dimensions of amplitude and phase, it is more capable of representing and handling uncertainty in the fusion process [30]. In particular, when CBBAs reduce to classical BBAs, CET degrades into DSET in the condition that the conflict coefficient is less than one. Consequently, CET affords a more generalized framework compared with the classical DSET.

In the classical DSET, distance plays an important role to measure the differences among BBAs, which is a benefit for conflict management. Many researchers dedicated much effort to address this problem in the past few decades [31,32], especially for the familiar distance of Jousselme et al. [33]. Subsequently, Jousselme and Maupin [34] provided a comprehensive survey on evidential distances. Later on, Bouchard et al. [35] proved a strict distance metric of Jousselme et al.’s distance. On the other hand, some scholars studied the difference measure from other perspectives, such as the correlation coefficient, and other hybrid models. For example, Jiang [36], Xiao [37], and Pan and Deng [38] researched the correlation coefficients among BBAs. Liu [39] analyzed the conflict degree by means of the conflict coefficient and distance among the betting commitments of BBAs. Even if the existing methods can well manage conflict problems in the classical DSET, very few of them have the ability to measure the difference among CBBAs in the complex plane framework of CET, except for the conflict coefficient [28,29] and complex evidential distance [40] of CET.

In this paper, inspired by Liu’s distance among the betting commitments of BBAs [39], a generalized betting commitment-based distance (BCD) is proposed to measure the difference among CBBAs in CET. To be specific, a complex Pignistic transformation is first proposed for not only singletons, but also subsets of CBBAs. Next, a betting commitment function is designed for all subsets of CBBAs on the basis of complex Pignistic transformation. Based on that, the distance among the betting commitments of CBBAs is devised to measure the difference among CBBAs. In particular, when CBBAs reduce to the classical BBAs, the BCD degenerates into Liu’s distance. Therefore, the proposed BCD is a generalized model to offer more capacity for measuring the difference among CBBAs. Additionally, other properties of the BCD are analyzed, including the non-negativeness, nondegeneracy, symmetry, and triangle inequality. It is then proven that the BCD is a strict distance metric because it satisfies distance axioms. Furthermore, BCD is compared with other related well-known methods to show its superiority. Besides, a basis algorithm and its weighted extension for multi-attribute decision-making are designed based on the newly defined BCD. Finally, these decision-making algorithms are applied to cope with the medical diagnosis problem under the smart IoT environment to reveal its effectiveness.

The contributions are summarized as follows:

- This is the first work to propose the complex pignistic transformation-based evidential betting commitment distance (BCD) for the multisource information fusion of medical diagnosis in the IoT.
- The BCD is a strict distance metric that satisfies the axioms of the nonnegativity, nondegeneracy, symmetry, and triangle inequality, which is a generalization of the classical evidential distance of Liu.
- A basis algorithm and its weighted extension for decision-making are designed on the basis of the BCD, which are applied to the medical IoT to demonstrate their effectiveness.

This paper is organized as follows. The preliminaries are introduced in Section 2. A new conflict measure model is proposed in Section 3. Section 4 provides several examples for the comparison and analysis of the proposed distance with other well-known methods. In Section 5, a basis multi-attribute decision-making algorithm and its extension are designed; then, they are applied to address the problem of medical diagnosis. Finally, Section 6 gives the conclusion.

## 2. Preliminaries

### 2.1. Medical IoT

A variety of publications have been presented to handle the problems of the medical IoT. The medical IoT technologies are mainly classified into two ares: remote monitoring and big data analysis [41].

For remote monitoring in the medical IoT, remote health checking and real-time location service are the key mechanisms. The sensor-based devices continuously record physiological signals with regard to the patient and then transfer the collected data to a monitoring server. Distant health care checking can be implemented via applications that get physiological data from patients. The collected data will be analyzed and processed according to smart algorithms to better support decision-making. Researchers have studied remote monitoring in the medical IoT from different perspectives. For example, Hossain and Muhammad [42] presented a health IoT-enabled monitoring framework, in which medical data are gathered through mobile devices and sensors and then securely transmitted to the cloud for seamless access by medical professionals. Gómez et al. [43] developed an architecture on the basis of an ontology to monitor health and workout routine recommendations to patients. Abawajy and Hassan [44] presented a pervasive patient health monitoring system infrastructure on the basis of integrated cloud computing and IoT technologies.

For big data analysis in the medical IoT, its purpose is to investigate and offer effective service. Due to accessing patient data both in routine clinical visits and at home, how to manage big data must be considered in accordance with data collection, data computing, analysis, and safety. Many researchers put forward various methods in this area. For instance, He and Zeadally [45] discussed the security requirements of RFID authentication schemes and presented a review of ECC-based RFID authentication schemes in terms of performance and security. Dimitrov [46] studied the medical IoT and big data in healthcare. Lomotey et al. [47] exploited an enhanced Petri nets service model to help for tracing medical data generation, tracking, and detecting data compromises. Zhang [48] devised a medical data fusion algorithm on the basis of the IoT. Dautov et al. [49] studied hierarchical data fusion for smart healthcare.

Through a careful analysis of existing publications, it is found that there is no research studying the data fusion problem of the medical IoT in the framework of complex evidence theory. Therefore, this work provides an alternative promising way to model and fuse medical data by means of complex evidence theory.

### 2.2. Uncertainty Modeling and Information Fusion

Multisource information fusion has received much attention in the past few years [50,51,52]. Because of the impacts of devices, the external environment, and communication problems, the collected information may be uncertain, imprecise, or even conflicting. How to handle such kinds of uncertainty is still an open issue [53,54]. So far, various theories and their corresponding methods have been exploited, such as the extended fuzzy probability [55], soft sets [56,57,58], interval numbers [59], evidence theory [60], Z numbers [61,62], complex distributions [63,64], complex intuitionistic fuzzy sets [65], quantum-based [66,67], and others [68,69], as well as the consensus measure [70]. These methods were applied in various fields, like medical diagnosis [71] and decision-making [72].

Among them, evidence theory provides a belief function to model uncertainty and Dempster’s rule of combination for the fusion of multisource information, so that it is effective in dealing with uncertain problems in information and has been applied in many areas [73], including classification [74,75], decision-making [76], and evaluation [77,78,79]. Specifically, CET inherits the merits of DSET and has a greater ability to represent and handle uncertainty in the fusion process, which will be introduced in the next section.

### 2.3. Complex Evidence Theory

The essential concepts of CET are introduced below [28,29].

**Definition 1.** *(Frame of discernment)*

*Let* Φ *be a frame of discernment (FOD), which is composed of:*

(1) $$\Phi =\{\phi_{1},\ldots ,\phi_{j},\ldots ,\phi_{n}\},$$

*where the elements in* Φ *are exclusive and collective, but not empty. The power set of* Φ *is expressed by:*

(2) $$\begin{array}{c} 2^{\Phi }=\left\{\emptyset ,\left\{\phi_{1}\right\},\left\{\phi_{2}\right\},\ldots ,\left\{\phi_{n}\right\},\left\{\phi_{1},\phi_{2}\right\},\ldots ,\left\{\phi_{1},\phi_{2},\ldots ,\phi_{i}\right\},\ldots ,\phi \right\}, \end{array}$$

*in which* ∅ *is the empty set.*

*If $A_{i}\in 2^{\Phi }$, $A_{i}$ is defined as a hypothesis, also called a proposition.*

**Definition 2.** *(Complex mass function)*

*A complex mass function (CMF), also called a CBBA, denoted as M in* Φ, *is defined as a mapping from $2^{\Phi }$ to C:*

(3) $$M:\;2^{\Phi }\to C$$

*satisfying:*

(4) $$\begin{array}{cc} \; & M(\emptyset )=0,\\ \; & M\left(A_{i}\right)=m\left(A_{i}\right)e^{i\theta (A_{i})},\;A_{i}\subseteq \Phi ,\\ \; & \sum_{A_{i}\in 2^{\Phi }}M\left(A_{i}\right)=1, \end{array}$$

*in which $i=\sqrt{-1}$, $m\left(A_{i}\right)\in \left[0,1\right]$ represents the magnitude of $M(A_{i})$ and $\theta \left(A_{i}\right)\in \left[-\pi ,\pi \right]$ represents a phase term.*

*In Equation (4), $M(A_{i})$ can also be expressed as:*

(5) $$M\left(A_{i}\right)=x+yi,\;A_{i}\subseteq \Phi$$

*and:*

(6) $$|M\left(A_{i}\right)|=m\left(A_{i}\right)=\sqrt{x^{2}+y^{2}},$$

*in which $\sqrt{x^{2}+y^{2}}\in \left[0,1\right]$.*

Note that the value of $\left|M(A_{i})\right|$ or $m(A_{i})$ represents the degree to which the evidence supports $A_{i}$.

**Definition 3.** *(Focal element)*

*If $\left|M(A_{i})\right|$ or $m(A_{i})>0$, $A_{i}$ is defined as a focal element.*

**Definition 4.** *(Complex Dempster’s rule of combination)*

*Let $M_{h}$ and $M_{k}$ be two independent CBBAs in* Φ*. The complex Dempster’s rule of combination (CDRC) is defined as $M=M_{u}⊕M_{v}$:*

(7) $$M\left(A_{k}\right)=\left\{\begin{array}{c} \begin{array}{cc} \frac{1}{1-K}\sum_{A_{i}\cap A_{h}=A_{k}}M_{u}\left(A_{i}\right)M_{v}\left(A_{h}\right), & \;A_{k}\neq \emptyset , \end{array}\\ \begin{array}{cc} 0, & \;A_{k}=\emptyset , \end{array} \end{array}\right.$$

*with:*

(8) $$K=\sum_{A_{i}\cap A_{h}=\emptyset }M_{u}\left(A_{i}\right)M_{v}\left(A_{h}\right),$$

*in which $A_{i},A_{h},A_{k}\in 2^{\Phi }$; K is the conflict coefficient between $M_{u}$ and $M_{v}$; |K| is used for the conflict measure between $M_{u}$ and $M_{v}$.*

### 2.4. |K| Versus Conflict

In this section, several numerical examples are provided to study the performance of |K| in CET for measuring the conflict. Specifically, in Examples 1 and 2, explicit explanations are given about how |K| expresses the conflict.

**Example** **1.** *Consider two CBBAs $M_{1}$ and $M_{2}$ in FOD $\Phi =\{\phi_{1},\phi_{2},\phi_{3},\phi_{4}\}$:*

$$\begin{array}{cc} M_{1}:\;\; & M_{1}\left(\left\{\phi_{1},\phi_{2}\right\}\right)=0.9055e^{i\arctan(0.1111)},\\ \; & M_{1}\left(\left\{\phi_{3}\right\}\right)=0.1414e^{i\arctan(-1.0000)},\\ \; & M_{1}\left(\left\{\phi_{4}\right\}\right)=0;\\ M_{2}:\;\; & M_{2}\left(\left\{\phi_{1},\phi_{2}\right\}\right)=0,\\ \; & M_{2}\left(\left\{\phi_{3}\right\}\right)=0.1414e^{i\arctan(-1.0000)},\\ \; & M_{2}\left(\left\{\phi_{4}\right\}\right)=0.9055e^{i\arctan(0.1111)}. \end{array}$$

By using Equation (8), the following is generated:

$$\begin{array}{c} |K|=1.0002. \end{array}$$

From the given CBBAs $M_{1}$ and $M_{2}$ in Example 1, it is noticed that $M_{1}$ and $M_{2}$ have stronger support degrees of 0.9055 to hypotheses $\left\{\phi_{1},\phi_{2}\right\}$ and $\left\{\phi_{4}\right\}$, respectively. Since the two hypotheses $\left\{\phi_{1},\phi_{2}\right\}$ and $\left\{\phi_{4}\right\}$ are incompatible, meaning that high conflict exists between $M_{1}$ and $M_{2}$. Hence, the value 1.0002 of |K| can effectively reflect the conflict between $M_{1}$ and $M_{2}$ in this example.

**Example** **2.** *Consider two CBBAs $M_{1}$ and $M_{2}$ in FOD $\Phi =\{\phi_{1},\phi_{2},\phi_{3}\}$:*

$$\begin{array}{cc} M_{1}:\;\; & M_{1}\left(\left\{\phi_{1}\right\}\right)=0.4123e^{i\arctan(0.2500)},\\ \; & M_{1}\left(\left\{\phi_{1},\phi_{2}\right\}\right)=0.4123e^{i\arctan(-0.2500)},\\ \; & M_{1}\left(\left\{\phi_{1},\phi_{2},\phi_{3}\right\}\right)=0.2;\\ M_{2}:\;\; & M_{2}\left(\left\{\phi_{1}\right\}\right)=0.4123e^{i\arctan(0.2500)},\\ \; & M_{2}\left(\left\{\phi_{1},\phi_{2}\right\}\right)=0.4123e^{i\arctan(-0.2500)},\\ \; & M_{2}\left(\left\{\phi_{1},\phi_{2},\phi_{3}\right\}\right)=0.2. \end{array}$$

By using Equation (8), the following is generated:

$$\begin{array}{c} |K|=0. \end{array}$$

From the given CBBAs $M_{1}$ and $M_{2}$ in Example 2, it is noticed that $M_{1}$ and $M_{2}$ have support degrees of 0.4123, 0.4123, and 0.2 to hypotheses $\left\{\phi_{1}\right\}$, $\left\{\phi_{1},\phi_{2}\right\}$, and $\left\{\phi_{1},\phi_{2},\phi_{3}\right\}$, respectively. Therefore, the result of |K| with the value of zero reflects nicely that $M_{1}$ and $M_{2}$ have completely the same beliefs.

Although |K| reflects the conflict between CBBAs very well in the above-discussed examples, it may not work in some certain situations. Example 3 illustrates such a case.

**Example** **3.** *Consider two CBBAs $M_{1}$ and $M_{2}$ in FOD $\Phi =\{\phi_{1},\phi_{2},\phi_{3},\phi_{4}\}$:*

$$\begin{array}{cc} M_{1}:\;\; & M_{1}\left(\left\{\phi_{1}\right\}\right)=\frac{1}{4},M_{1}\left(\left\{\phi_{2}\right\}\right)=\frac{1}{4},M_{1}\left(\left\{\phi_{3}\right\}\right)=\frac{1}{4},M_{1}\left(\left\{\phi_{4}\right\}\right)=\frac{1}{4};\\ M_{2}:\;\; & M_{2}\left(\left\{\phi_{1}\right\}\right)=\frac{1}{4},M_{2}\left(\left\{\phi_{2}\right\}\right)=\frac{1}{4},M_{2}\left(\left\{\phi_{3}\right\}\right)=\frac{1}{4},M_{2}\left(\left\{\phi_{4}\right\}\right)=\frac{1}{4}. \end{array}$$

By using Equation (8), the following is generated:

$$\begin{array}{c} |K|=0.75. \end{array}$$

From the given CBBAs $M_{1}$ and $M_{2}$ in Example 3, it can be seen that $M_{1}$ and $M_{2}$ have the same support degrees of $\frac{1}{4}$ to hypotheses $\left\{\phi_{1}\right\}$, $\left\{\phi_{2}\right\}$, $\left\{\phi_{3}\right\}$, and $\left\{\phi_{4}\right\}$, respectively. This means that $M_{1}$ and $M_{2}$ are completely the same as each other, so that the expected value of |K| should be zero. Therefore, the result of |K| with the value of 0.75 is counter-intuitive.

Consequently, this motivates designing a new conflict measure model for CBBAs in CET.

## 3. A New Conflict Measure Model

In this section, a complex Pignistic transformation is first defined. Based on the complex Pignistic transformation, a betting commitment function is proposed for all subsets of CBBAs. Then, a new distance model is designed by taking advantage of the betting commitment function. Furthermore, several corresponding examples are illustrated in terms of betting the commitment function.

### 3.1. Complex Pignistic Transformation

As discussed in Section 2.3, a complex mass function in CET is introduced. It is founded that making a decision is difficult on the basis of the complex belief function. Hence, a complex Pignistic transformation function [80] is proposed to transform the complex mass function to address this problem.

**Definition 5.** *(Complex Pignistic transformation for $\phi_{j}$)*

*Let M be a complex mass function on FOD* Φ *and $A_{i}$ be a hypothesis with $A_{i}\subseteq \Phi$. The complex Pignistic transformation function for $\phi_{j}$ on* Φ *is defined by:*

(9) $$CPT\left(\phi_{j}\right)=\sum_{A_{i}\subseteq \Phi ,\phi_{j}\in A_{i}}\frac{M(A_{i})}{|A_{i}|},\;\forall \phi_{j}\in \Phi ,$$

*where $|A_{i}|$ denotes the number of elements in $A_{i}$.*

In accordance with Definition 5, a complex Pignistic transformation function for $A_{i}$ on $2^{\Phi }$ is defined below.

**Definition 6.** *(Complex Pignistic transformation for $A_{i}$)*

*A complex Pignistic transformation function for $A_{i}$ on $2^{\Phi }$ is defined as:*

(10) $$CPT\left(A_{i}\right)=\sum_{\phi_{j}\in A_{i}}CPT\left(\phi_{j}\right).$$

**Corollary** **1.** *If M is a probability distribution P, then CPT is equal to P.*

Then, Equations (9) and (10) can be integrated as in Definition 7.

**Definition 7.** *(Complex Pignistic transformation)*

*Let $M^{\Phi }$ be a complex mass function on FOD* Φ*. The complex Pignistic transformation function is defined by:*

(11) $$CPT\left(A_{h}\right)=\sum_{A_{i},A_{h}\subseteq \Phi }M\left(A_{i}\right)\frac{|A_{i}\cap A_{h}|}{|A_{h}|},$$

*where $|A_{i}\cap A_{h}|$ represents the number of elements in the intersection $A_{i}\cap A_{h}$; $|A_{h}|$ denotes the number of elements in $A_{h}$.*

Based on Definition 7, a betting commitment function is defined to $A_{h}$ ($A_{h}\subseteq \Phi$).

**Definition 8.** *(Betting commitment)*

*The betting commitment to $A_{h}$ on FOD* Φ *is defined by:*

(12) $$BetC\left(A_{h}\right)=\left|CPT(A_{h})\right|,\;\forall A_{h}\subseteq \Phi ,$$

*where |·| denotes the absolute value function.*

### 3.2. Betting Commitment-Based Distance Versus Conflict

In the following Examples 4–6, explicit explanations are given about how the betting commitment to express the conflict.

**Example** **4.** *Consider two CBBAs $M_{1}$ and $M_{2}$ defined in Example 1.*

By using Equation (12), the following is generated:

$$\begin{array}{cc} BetC_{M_{1}}\left(\left\{\phi_{1},\phi_{2}\right\}\right) & =0.9055,\\ BetC_{M_{1}}\left(\left\{\phi_{3}\right\}\right) & =0.1414,\\ BetC_{M_{1}}\left(\left\{\phi_{4}\right\}\right) & =0.0000; \end{array}$$

and

$$\begin{array}{cc} BetC_{M_{2}}\left(\left\{\phi_{1},\phi_{2}\right\}\right) & =0.0000,\\ BetC_{M_{2}}\left(\left\{\phi_{3}\right\}\right) & =0.1414,\\ BetC_{M_{2}}\left(\left\{\phi_{4}\right\}\right) & =0.9055. \end{array}$$

Through calculating the absolute value of the difference between $BetC_{M_{1}}$ and $BetC_{M_{2}}$ in Example 4, we get:

$$\begin{array}{c} |BetC_{M_{1}}\left(\left\{\phi_{1},\phi_{2}\right\}\right)-BetC_{M_{2}}\left(\left\{\phi_{1},\phi_{2}\right\}\right)|=0.9055,\\ |BetC_{M_{1}}\left(\left\{\phi_{3}\right\}\right)-BetC_{M_{2}}\left(\left\{\phi_{3}\right\}\right)|=0.0000,\\ |BetC_{M_{1}}\left(\left\{\phi_{4}\right\}\right)-BetC_{M_{2}}\left(\left\{\phi_{4}\right\}\right)|=0.9055. \end{array}$$

On the other hand, by using Equation (8), the following is generated:

$$\begin{array}{c} |K|=1.0002, \end{array}$$

which indicates the significant discrepancy between CBBAs $M_{1}$ and $M_{2}$ that satisfies the intuitive result.

In this context, $|BetC_{M_{1}}\left(\left\{\phi_{1},\phi_{2}\right\}\right)-BetC_{M_{2}}\left(\left\{\phi_{1},\phi_{2}\right\}\right)|$ and $|BetC_{M_{1}}\left(\left\{\phi_{4}\right\}\right)-$$BetC_{M_{2}}\left(\left\{\phi_{4}\right\}\right)|$ have the maximal value of 0.9055, which also reflects the discrepancy between CBBAs $M_{1}$ and $M_{2}$ significantly.

**Example** **5.** *Consider two CBBAs $M_{1}$ and $M_{2}$ defined in Example 2.*

By using Equation (12), the following is generated:

$$\begin{array}{cc} BetC_{M_{1}}\left(\left\{\phi_{1},\phi_{2}\right\}\right) & =0.6685,\\ BetC_{M_{1}}\left(\left\{\phi_{3}\right\}\right) & =0.9333,\\ BetC_{M_{1}}\left(\left\{\phi_{4}\right\}\right) & =1.0000; \end{array}$$

and

$$\begin{array}{cc} BetC_{M_{2}}\left(\left\{\phi_{1},\phi_{2}\right\}\right) & =0.6685,\\ BetC_{M_{2}}\left(\left\{\phi_{3}\right\}\right) & =0.9333,\\ BetC_{M_{2}}\left(\left\{\phi_{4}\right\}\right) & =1.0000. \end{array}$$

Through calculating the absolute value of the difference between $BetC_{M_{1}}$ and $BetC_{M_{2}}$ in Example 4, we get:

$$\begin{array}{c} |BetC_{M_{1}}\left(\left\{\phi_{1},\phi_{2}\right\}\right)-BetC_{M_{2}}\left(\left\{\phi_{1},\phi_{2}\right\}\right)|=0,\\ |BetC_{M_{1}}\left(\left\{\phi_{3}\right\}\right)-BetC_{M_{2}}\left(\left\{\phi_{3}\right\}\right)|=0,\\ |BetC_{M_{1}}\left(\left\{\phi_{4}\right\}\right)-BetC_{M_{2}}\left(\left\{\phi_{4}\right\}\right)|=0. \end{array}$$

On the other hand, by using Equation (8), the following is generated:

$$\begin{array}{c} |K|=0, \end{array}$$

which indicates that CBBAs $M_{1}$ and $M_{2}$ have completely the same beliefs satisfying the intuitive result.

In this context, $|BetC_{M_{1}}\left(\left\{\phi_{1},\phi_{2}\right\}\right)-BetC_{M_{2}}\left(\left\{\phi_{1},\phi_{2}\right\}\right)|$, $|BetC_{M_{1}}\left(\left\{\phi_{3}\right\}\right)-BetC_{M_{2}}\left(\left\{\phi_{3}\right\}\right)|$, and $|BetC_{M_{1}}\left(\left\{\phi_{4}\right\}\right)-$$BetC_{M_{2}}\left(\left\{\phi_{4}\right\}\right)|$ have the minimal value of zero, which also reflects well that CBBAs $M_{1}$ and $M_{2}$ have completely the same beliefs.

**Example** **6.** *Consider two CBBAs $M_{1}$ and $M_{2}$ defined in Example 3.*

By using Equation (12), the following is generated:

$$\begin{array}{c} BetC_{M_{1}}\left(\left\{\phi_{1}\right\}\right)=0.25,\\ BetC_{M_{1}}\left(\left\{\phi_{2}\right\}\right)=0.25,\\ BetC_{M_{1}}\left(\left\{\phi_{3}\right\}\right)=0.25,\\ BetC_{M_{1}}\left(\left\{\phi_{4}\right\}\right)=0.25; \end{array}$$

and:

$$\begin{array}{c} BetC_{M_{2}}\left(\left\{\phi_{1}\right\}\right)=0.25,\\ BetC_{M_{2}}\left(\left\{\phi_{2}\right\}\right)=0.25,\\ BetC_{M_{2}}\left(\left\{\phi_{3}\right\}\right)=0.25,\\ BetC_{M_{2}}\left(\left\{\phi_{4}\right\}\right)=0.25. \end{array}$$

Through calculating the absolute value of the difference between $BetC_{M_{1}}$ and $BetC_{M_{2}}$ in Example 6, we get:

$$\begin{array}{c} |BetC_{M_{1}}\left(\left\{\phi_{1},\phi_{2}\right\}\right)-BetC_{M_{2}}\left(\left\{\phi_{1},\phi_{2}\right\}\right)|=0,\\ |BetC_{M_{1}}\left(\left\{\phi_{3}\right\}\right)-BetC_{M_{2}}\left(\left\{\phi_{3}\right\}\right)|=0,\\ |BetC_{M_{1}}\left(\left\{\phi_{4}\right\}\right)-BetC_{M_{2}}\left(\left\{\phi_{4}\right\}\right)|=0. \end{array}$$

On the other hand, by using Equation (8), the following is generated:

$$\begin{array}{c} |K|=0.75, \end{array}$$

which indicates the significant discrepancy between CBBAs $M_{1}$ and $M_{2}$.

However, as discussed in Section 2.4, this result of |K| in Example 6 is count-intuitive, since all the belief values of the focal elements of $M_{1}$ and $M_{2}$ are exactly the same.

Nevertheless, the values of $|BetC_{M_{1}}\left(\left\{\phi_{1},\phi_{2}\right\}\right)-BetC_{M_{2}}\left(\left\{\phi_{1},\phi_{2}\right\}\right)|$, $|BetC_{M_{1}}\left(\left\{\phi_{3}\right\}\right)$$-BetC_{M_{2}}\left(\left\{\phi_{3}\right\}\right)|$, and $|BetC_{M_{1}}\left(\left\{\phi_{4}\right\}\right)-BetC_{M_{2}}\left(\left\{\phi_{4}\right\}\right)|$ are equal to zero, which reflects that there is no discrepancy between CBBAs $M_{1}$ and $M_{2}$. Therefore, this distance measure among the betting commitments of CBBAs satisfies our expectation.

Consequently, from Examples 4–6, it is learned that the distance model based on the betting commitment has a better performance for measuring conflict comparing with the classical |K| in CET. Taking this into consideration, a new distance measure is designed on the basis of the betting commitment function, which is defined below.

**Definition 9.** *(Betting commitment-based distance of CBBAs)*

*Let $M_{u}$ and $M_{v}$ be two CBBAs on FOD* Φ *and $A_{i}$ be a hypothesis with $A_{i}\subseteq \Phi$. The distance between betting commitments of CBBAs, called BCD, is defined by:*

(13) $$d_{BCD}\left(M_{u},M_{v}\right)=\max_{A_{i}\subseteq \Phi }\left\{|BetC_{M_{u}}\left(A_{i}\right)-BetC_{M_{v}}\left(A_{i}\right)|\right\},$$

*where |·| denotes the absolute value function.*

In Equation (13), the maximal differences among the betting commitments of CBBAs for all subsets are taken into account. The reason is that considering the conflict measure in Example 4, min or mean functions are not adequate to distinguish the difference among CBBAs compared with the max function.

In particular, when CBBAs $M_{u}$ and $M_{v}$ reduce to the classical BBAs $m_{u}$ and $m_{v}$, $M_{u}=m_{u}$ and $M_{v}=m_{v}$. Since:

$$\begin{array}{cc} d_{BCD}\left(M_{u},M_{v}\right)\; & =\max_{A_{i}\subseteq \Phi }\left\{|BetC_{M_{u}}\left(A_{i}\right)-BetC_{M_{v}}\left(A_{i}\right)|\right\}\\ \; & =\max_{A_{i}\subseteq \Phi }\left\{||CPT_{M_{u}}\left(A_{i}\right)|-|CPT_{M_{v}}\left(A_{i}\right)\left|\right|\right\}\\ \; & =\max_{A_{i}\subseteq \Phi }\left\{\left|\left|\sum_{A_{i},A_{h}\subseteq \Phi }M\left(A_{i}\right)\frac{|A_{i}\cap A_{h}|}{|A_{h}|}\right|-\left|\sum_{A_{i},A_{h}\subseteq \Phi }M\left(A_{i}\right)\frac{|A_{i}\cap A_{h}|}{|A_{h}|}\right|\right|\right\}, \end{array}$$

we have:

(14) $$\begin{array}{cc} d_{BCD}\left(M_{u},M_{v}\right)\; & =\max_{A_{i}\subseteq \Phi }\left\{\left|\left|\sum_{A_{i},A_{h}\subseteq \Phi }m\left(A_{i}\right)\frac{|A_{i}\cap A_{h}|}{|A_{h}|}\right|-\left|\sum_{A_{i},A_{h}\subseteq \Phi }m\left(A_{i}\right)\frac{|A_{i}\cap A_{h}|}{|A_{h}|}\right|\right|\right\}\\ \; & =\max_{A_{i}\subseteq \Phi }\left\{\left|\sum_{A_{i},A_{h}\subseteq \Phi }m\left(A_{i}\right)\frac{|A_{i}\cap A_{h}|}{|A_{h}|}-\sum_{A_{i},A_{h}\subseteq \Phi }m\left(A_{i}\right)\frac{|A_{i}\cap A_{h}|}{|A_{h}|}\right|\right\}. \end{array}$$

In this case that $M_{u}=m_{u}$ and $M_{v}=m_{v}$, Equation (14) can be expressed as:

(15) $$\begin{array}{c} d_{BCD}\left(M_{u},M_{v}\right)=\max_{A_{i}\subseteq \Phi }\left\{|BetC_{m_{u}}\left(A_{i}\right)-BetC_{m_{v}}\left(A_{i}\right)|\right\}, \end{array}$$

which is obviously the same as Liu’s distance measure difBetP [39]. This means that when the CBBAs become the classical BBAs, the proposed BCD $d_{BCD}$ degenerates into Liu’s distance measure difBetP.

**Theorem** **1.** *The BCD $d_{BCD}$ is a generalized model of the traditional distance measure of Liu’s difBetP [39].*

**Theorem** **2.** *The BCD $d_{BCD}$ is a strict distance metric.*

**Property** **1.** *Consider three arbitrary CBBAs: $M_{u}$, $M_{v}$, and $M_{w}$. BCD $d_{BCD}$ holds the following properties:*

*P1.1 Nonnegativity: $d_{BCD}\left(M_{u},M_{v}\right)\geq 0$.*

*P1.2 Nondegeneracy: $d_{BCD}\left(M_{u},M_{v}\right)=0$ if and only if $M_{u}=M_{v}$.*

*P1.3 Symmetry: $d_{BCD}\left(M_{u},M_{v}\right)$ = $d_{BCD}\left(M_{v},M_{u}\right)$.*

*P1.4 Triangle inequality: $d_{BCD}\left(M_{u},M_{w}\right)\leq d_{BCD}\left(M_{u},M_{v}\right)+d_{BCD}\left(M_{v},M_{w}\right)$.*

**Proof.** (1) Consider two arbitrary CBBAs: $M_{u}$ and $M_{v}$. According to Equation (13), it is obvious that $d_{BCD}\left(M_{u},M_{v}\right)\geq 0$, because of having the absolute value function.

(2) Consider two arbitrary CBBAs: $M_{u}=M_{v}$; we get:

$$\begin{array}{c} d_{BCD}\left(M_{u},M_{v}\right)=\max_{A_{i}\subseteq \Phi }\left\{|BetC_{M_{u}}\left(A_{i}\right)-BetC_{M_{u}}\left(A_{i}\right)|\right\}=0. \end{array}$$

Next, consider $d_{BCD}\left(M_{u},M_{v}\right)=0$, then:

$$\begin{array}{c} \max_{A_{i}\subseteq \Phi }\left\{|BetC_{M_{u}}\left(A_{i}\right)-BetC_{M_{u}}\left(A_{i}\right)|\right\}=0. \end{array}$$

Thus, for $\forall A_{i}\subseteq \Phi$, we obtain:

$$\begin{array}{c} M_{u}\left(A_{i}\right)=M_{v}\left(A_{i}\right). \end{array}$$

Hence, it is proven that $d_{BCD}\left(M_{u},M_{v}\right)=0⟺M_{u}=M_{v}$.

(3) Consider two arbitrary CBBAs $M_{u}$ and $M_{v}$; we have $d_{BCD}\left(M_{v},M_{u}\right)$:

$$\begin{array}{c} d_{BCD}\left(M_{u},M_{v}\right)=\max_{A_{i}\subseteq \Phi }\left\{|BetC_{M_{u}}\left(A_{i}\right)-BetC_{M_{u}}\left(A_{i}\right)|\right\} \end{array}$$

Consider $d_{BCD}\left(M_{v},M_{u}\right)$; we have:

$$\begin{array}{c} d_{BCD}\left(M_{v},M_{u}\right)=\max_{A_{i}\subseteq \Phi }\left\{|BetC_{M_{v}}\left(A_{i}\right)-BetC_{M_{u}}\left(A_{i}\right)|\right\} \end{array}$$

Thus, we obtain that:

$$\begin{array}{c} d_{BCD}\left(M_{u},M_{v}\right)=d_{BCD}\left(M_{v},M_{u}\right), \end{array}$$

which proves the property of symmetry.

(4) Consider three arbitrary CBBAs: $M_{u}$, $M_{v}$, and $M_{w}$.

From the triangle inequality for real numbers, we have:

$$\begin{array}{c} |BetC_{M_{u}}\left(A_{i}\right)-BetC_{M_{w}}\left(A_{i}\right)|\leq |BetC_{M_{u}}\left(A_{i}\right)-BetC_{M_{v}}\left(A_{i}\right)|+|BetC_{M_{v}}\left(A_{i}\right)-BetC_{M_{w}}\left(A_{i}\right)|, \end{array}$$

Through the nature of the max operation, we obtained that:

$$\begin{array}{c} |BetC_{M_{u}}\left(A_{i}\right)-BetC_{M_{v}}\left(A_{i}\right)|\leq \max_{A_{i}\subseteq \Phi }\{|BetC_{M_{u}}\left(A_{i}\right)-BetC_{M_{v}}\left(A_{i}\right)\left|\right\}, \end{array}$$

and:

$$\begin{array}{c} |BetC_{M_{v}}\left(A_{i}\right)-BetC_{M_{w}}\left(A_{i}\right)|\leq \max_{A_{i}\subseteq \Phi }\{|BetC_{M_{v}}\left(A_{i}\right)-BetC_{M_{w}}\left(A_{i}\right)\left|\right\}. \end{array}$$

Thus,

$$\begin{array}{c} |BetC_{M_{u}}\left(A_{i}\right)-BetC_{M_{v}}\left(A_{i}\right)|+|BetC_{M_{v}}\left(A_{i}\right)-BetC_{M_{w}}\left(A_{i}\right)|\leq \\ \max_{A_{i}\subseteq \Phi }\{|BetC_{M_{u}}\left(A_{i}\right)-BetC_{M_{v}}\left(A_{i}\right)\left|\right\}+\max_{A_{i}\subseteq \Phi }\{|BetC_{M_{v}}\left(A_{i}\right)-BetC_{M_{w}}\left(A_{i}\right)\left|\right\}. \end{array}$$

Therefore,

$$\begin{array}{c} d_{BCD}\left(M_{u},M_{w}\right)\leq d_{BCD}\left(M_{u},M_{v}\right)+d_{BCD}\left(M_{v},M_{w}\right). \end{array}$$

 □

## 4. Comparisons and Analysis

In this section, the proposed conflict measure BCD is compared with other well-known methods of |K| [28,29], $d_{CBBA}$ [40], and difBetP [39]. In addition, several examples are provided to illustrate their performance in terms of the conflict measure.

**Example** **7.** *Consider two CBBAs: $M_{1}$ and $M_{2}$, in $\Phi =\{\phi_{1},\phi_{2},\phi_{3},\ldots ,\phi_{20}\}$:*

$$\begin{array}{cc} M_{1}: & \;M_{1}\left(\left\{\phi_{2},\phi_{3},\phi_{4}\right\}\right)=\sqrt{0.5^{2}+\alpha^{2}}e^{i\arctan(\frac{\alpha }{0.5})},M_{1}\left(\left\{\phi_{7}\right\}\right)=0.05,\\ \; & \;M_{1}\left(\Phi \right)=0.1,M_{1}\left(A_{i}\right)=\sqrt{0.8^{2}+\alpha^{2}}e^{i\arctan(-\frac{\alpha }{0.8})};\\ M_{2}: & \;M_{2}\left(\left\{\phi_{1},\phi_{2},\phi_{3},\phi_{4},\phi_{5}\right\}\right)=1. \end{array}$$

**Figure 1:**
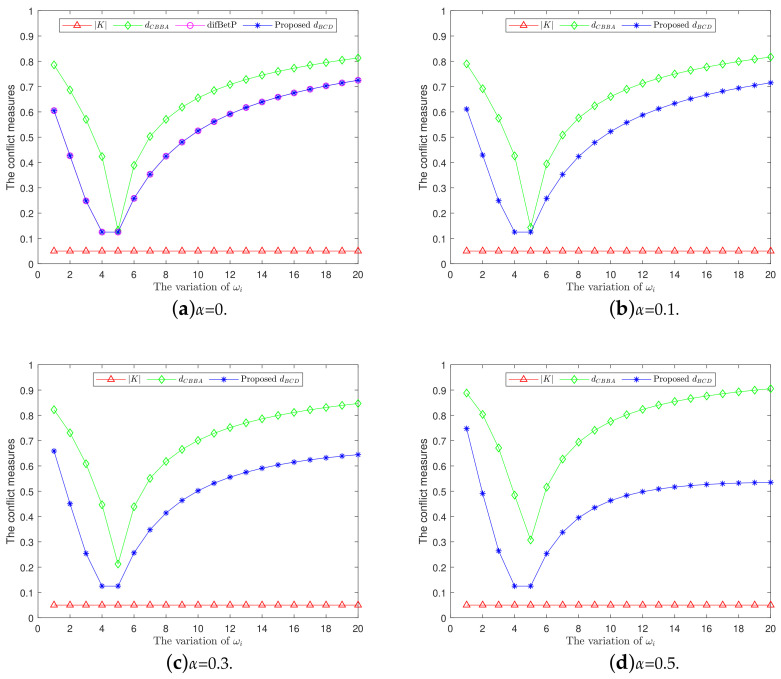
The conflict measures in Example 7.

In Example 7, hypothesis $A_{i}$ of $M_{1}$ changes from $\left\{\phi_{1}\right\}$ to Φ as in Table 1. To be specific, $M_{1}$ has four focal elements: $M_{1}\left(\left\{\phi_{2},\phi_{3},\phi_{4}\right\}\right)$, $M_{1}\left(\left\{\phi_{7}\right\}\right)$, $M_{1}\left(\Phi \right)$, and $M_{1}\left(A_{i}\right)$. According to Equation (6) in Definition 2, $\sqrt{0.8^{2}+\alpha^{2}}$ must be less than or equal to one. Therefore, α is set as 0, 0.1, 0.3, to 0.5 here, and the values of $M_{1}\left(\left\{\phi_{2},\phi_{3},\phi_{4}\right\}\right)$ and $M_{1}\left(A_{i}\right)$ change with α. $M_{2}$ has one focal element: $M_{2}\left(\left\{A,B,C,D,E\right\}\right)=1$. Through utilizing |K|, $d_{CBBA}$, difBetP, and the proposed $d_{BCD}$, their corresponding conflict measures between CBBAs $M_{1}$ and $M_{2}$ are depicted in Figure 1.

When α=0, this means that CBBA $M_{1}$ reduces to a classical BBA. From Figure 1a, it is clear that $d_{BCD}$ has exactly the same measure as difBetP; both of them have the same measure trends as $d_{CBBA}$. In particular, as the hypothesis $A_{i}$ of $M_{1}$ increases from $\left\{\phi_{1}\right\}$ to $\left\{\phi_{1},\phi_{2},\phi_{3},\phi_{4},\phi_{5}\right\}$, the measure values of $d_{CBBA}$, difBetP, and $d_{BCD}$ go down. When $A_{i}=\left\{\phi_{1},\phi_{2},\phi_{3},\phi_{4},\phi_{5}\right\}$, the conflict measures of $d_{CBBA}$, difBetP, and $d_{BCD}$ achieve their minimal value. Subsequently, as hypothesis $A_{i}$ adds from $\left\{\phi_{1},\phi_{2},\phi_{3},\phi_{4},\phi_{5}\right\}$ to Φ, the conflict measures of $d_{CBBA}$, difBetP and $d_{BCD}$ get larger and larger. However, the conflict measure of |K| remains the same regardless of the change of hypothesis $A_{i}$.

On the other hand, Figure 1b–d depicts how the conflict measures of different methods change with the variations of α=0.1, α=0.3, and α=0.5, respectively. It is obvious that no matter whether α=0.1, α=0.3, or α=0.5, $d_{CBBA}$ and $d_{BCD}$ can effectively measure the conflict between CBBAs $M_{1}$ and $M_{2}$. Note that the conflict value of $d_{CBBA}$ is always larger than that of $d_{BCD}$. The reason is that $d_{BCD}$ selects the maximal value of the difference between betting commitments to all subsets, but $d_{CBBA}$ is a kind of accumulating distance measure. Nevertheless, difBetP cannot measure the conflict among CBBAs, while the conflict measure of |K| also remains the same despite the change of $A_{i}$.

It can then be concluded that the methods of $d_{CBBA}$ and $d_{BCD}$ have better performance to measure the conflict among CBBAs than the difBetP and |K| methods.

**Example** **8.** Consider two CBBAs $M_{1}$ and $M_{2}$ in FOD $\Phi =\{\phi_{1},\phi_{2},\phi_{3},\phi_{4},\phi_{5}\}$:

$$\begin{array}{cc} \mathrm{Case}\;1:\;M_{1}^{C_{1}}:\;\; & M_{1}^{C_{1}}\left(\left\{\phi_{1},\phi_{2}\right\}\right)=0.8062e^{i\arctan(0.1250)},\\ \; & M_{1}^{C_{1}}\left(\left\{\phi_{3}\right\}\right)=0.1414e^{i\arctan(-1.0000)},\\ \; & M_{1}^{C_{1}}\left(\left\{\phi_{4}\right\}\right)=0.1;\\ M_{2}^{C_{1}}:\;\; & M_{2}^{C_{1}}\left(\left\{\phi_{1},\phi_{2}\right\}\right)=0.1,\\ \; & M_{2}^{C_{1}}\left(\left\{\phi_{3}\right\}\right)=0.1414e^{i\arctan(-1.0000)},\\ \; & M_{2}^{C_{1}}\left(\left\{\phi_{4}\right\}\right)=0.8062e^{i\arctan(0.1250)}.\\ \mathrm{Case}\;2:\;M_{1}^{C_{2}}:\;\; & M_{1}^{C_{2}}\left(\left\{\phi_{1},\phi_{2},\phi_{4}\right\}\right)=0.8062e^{i\arctan(0.1250)},\\ \; & M_{1}^{C_{2}}\left(\left\{\phi_{3}\right\}\right)=0.1414e^{i\arctan(-1.0000)},\\ \; & M_{1}^{C_{2}}\left(\left\{\phi_{4}\right\}\right)=0.1;\\ M_{2}^{C_{2}}:\;\; & M_{2}^{C_{2}}\left(\left\{\phi_{1},\phi_{2}\right\}\right)=0.1,\\ \; & M_{2}^{C_{2}}\left(\left\{\phi_{3}\right\}\right)=0.1414e^{i\arctan(-1.0000)},\\ \; & M_{2}^{C_{2}}\left(\left\{\phi_{4}\right\}\right)=0.8062e^{i\arctan(0.1250)}.\\ \mathrm{Case}\;3:\;M_{1}^{C_{3}}:\;\; & M_{1}^{C_{3}}\left(\left\{\phi_{1}\right\}\right)=0.8062e^{i\arctan(0.1250)},\\ \; & M_{1}^{C_{3}}\left(\left\{\phi_{2},\phi_{3},\phi_{4},\phi_{5}\right\}\right)=0.2236e^{i\arctan(-0.5000)};\\ M_{2}^{C_{3}}:\;\; & M_{2}^{C_{3}}\left(\Phi \right)=1. \end{array}$$

In Example 8, comparing the CBBAs of Case 1 with the CBBAs of Case 2 and Case 3, it is noticed that $M_{1}^{C_{1}}$ and $M_{2}^{C_{1}}$ are more conflicting with regard to their corresponding belief values than $M_{1}^{C_{2}}$ and $M_{2}^{C_{1}}$, or $M_{1}^{C_{3}}$ and $M_{2}^{C_{3}}$. This is because $M_{1}^{C_{1}}$ has a stronger belief value of 0.8062 to support $\left\{\phi_{1},\phi_{2}\right\}$, but $M_{2}^{C_{1}}$ has a stronger belief value of 0.8062 to support $\left\{\phi_{4}\right\}$. Since $\left\{\phi_{1},\phi_{2}\right\}$ and $\left\{\phi_{4}\right\}$ are incompatible, in accordance with our expectation, the conflict measure between $M_{1}^{C_{1}}$ and $M_{2}^{C_{1}}$ is supposed to be larger than that of $M_{1}^{C_{2}}$ and $M_{2}^{C_{2}}$, or $M_{1}^{C_{3}}$ and $M_{2}^{C_{3}}$.

By utilizing |K|, $d_{CBBA}$, and $d_{BCD}$, the three cases corresponding to the conflict measures among CBBAs: $M_{1}^{C_{1}}$ and $M_{2}^{C_{1}}$, $M_{1}^{C_{2}}$ and $M_{2}^{C_{2}}$, and $M_{1}^{C_{3}}$ and $M_{2}^{C_{3}}$ are measured as described in Table 2, respectively. Through careful analysis, the following interesting results are found:

R 1: For |K|, it can be seen that it can measure well the conflict between CBBAs in Case 1 and Case 2 with values of 0.84 and 0.264, respectively. However, |K| cannot distinguish the difference among the CBBAs in Case 3 with a measure value of zero.
R 2: For $d_{CBBA}$, it is obvious that $d_{CBBA}$ can measure the conflicts among the CBBAs in Case 1, Case 2, and Case 3 with values of 0.7071, 0.5802, and 0.7242, respectively. Comparing the conflict value of 0.7071 between $M_{1}^{C_{1}}$ and $M_{2}^{C_{1}}$ and 0.7242 between $M_{1}^{C_{3}}$ and $M_{2}^{C_{3}}$, it is noticed that the result of $d_{CBBA}\left(M_{1}^{C_{3}},M_{2}^{C_{3}}\right)>d_{CBBA}\left(M_{1}^{C_{1}},M_{2}^{C_{1}}\right)$ is not up to our expectations.
R 3: For $d_{BCD}$, it is easy to see that $d_{BCD}$ can well measure the conflicts among the CBBAs in all three cases with values of 0.7062, 0.4380, and 0.6062, respectively. Comparing the conflict value of 0.7062 of $d_{BCD}\left(M_{1}^{C_{1}},M_{2}^{C_{1}}\right)$ with 0.6062 of $d_{BCD}\left(M_{1}^{C_{3}},M_{2}^{C_{3}}\right)$, it is obtained that $d_{BCD}\left(M_{1}^{C_{3}},M_{2}^{C_{3}}\right)<d_{BCD}\left(M_{1}^{C_{1}},M_{2}^{C_{1}}\right)$. This result satisfies our expectation.
R 4: It is concluded that the BCD $d_{BCD}$ is a better conflict measure compared with the methods of |K| and $d_{CBBA}$ to judge the contradiction among CBBAs.

## 5. Algorithm and Application

Multi-attribute decision-making has received much attention [81,82,83]. Because of the complexity of various applications, it is still an open issue to handle uncertainty in this field. In this section, a basis algorithm for multi-attribute decision-making is first designed based on the newly defined BCD. After that, the proposed basis algorithm is applied to deal with a medical diagnosis decision-making problem to show its feasibility. Furthermore, this basis algorithm is extended to a weighted scheme to better fit real applications by taking into consideration different weights with regard to multiple attributes. Finally, both the basis and weighted algorithms are compared with well-known related methods to demonstrate their effectiveness.

### 5.1. Algorithm for Decision-Making

Problem statement: Let X be a set of attributes: $\left\{a_{1},\ldots ,a_{\kappa },\ldots ,a_{\eta }\right\}$ and P be a set of patterns: $\left\{p_{1},\ldots ,p_{j},\ldots ,p_{g}\right\}$ modeled by CBBA $p_{j}=\{\langle a_{\kappa },M_{p_{j}}^{a_{\kappa }}\left(\left\{y\right\}\right),$ $M_{p_{j}}^{a_{\kappa }}\left(\left\{n\right\}\right),M_{p_{j}}^{a_{\kappa }}\left(\left\{y,n\right\}\right)\rangle |a_{\kappa }\in X\}$. Consider a set of samples: $S=\{s_{1},\ldots ,s_{h},\ldots ,s_{l}\}$ modeled by CBBA $s_{h}=\left\{\langle a_{\kappa },M_{s_{h}}^{a_{\kappa }}\left(\left\{y\right\}\right),M_{s_{h}}^{a_{\kappa }}\left(\left\{n\right\}\right),M_{s_{h}}^{a_{\kappa }}\left(\left\{y,n\right\}\right)\rangle |a_{\kappa }\in X\right\}$. This algorithm for multi-attribute decision-making sorts the samples according to the given patterns.

Step 1: The BCD $d_{BCD}$ is used to calculate the distance between $s_{h}$ and $p_{j}$:
  (16) $$\begin{array}{cc} d_{BCD}\left(M_{s_{h}},M_{p_{j}}\right)\; & =\frac{1}{\eta }\sum_{\kappa =1}^{\eta }d_{BCD}\left(M_{s_{h}}^{a_{\kappa }},M_{p_{j}}^{a_{\kappa }}\right)\\ \; & =\frac{1}{\eta }\sum_{\kappa =1}^{\eta }\max_{A_{i}\subseteq \Phi }\left\{\left|BetC_{M_{s_{h}}^{a_{\kappa }}}\left(A_{i}\right)-BetC_{M_{p_{j}}^{a_{\kappa }}}\left(A_{i}\right)\right|\right\}. \end{array}$$
Step 2: The minimal distance between $M_{s_{h}}$ and $M_{p_{j}}$ is elected:
  (17) $$d_{BCD}\left(M_{s_{h}},M_{p_{\theta }}\right)=\min_{1\leq j\leq g}d_{BCD}\left(M_{s_{h}},M_{p_{j}}\right).$$
Step 3: $s_{h}$ is sorted into pattern $p_{\theta }$ by:
  (18) $$\begin{array}{cc} \; & \theta =\underset{1\leq j\leq m}{\arg\min}\left\{d_{BCD}\left(M_{s_{h}},M_{p_{j}}\right)\right\},\\ \; & s_{h}\leftarrow p_{\theta }. \end{array}$$

The corresponding pseudo-code of multi-attribute decision-making is described in Algorithm 1.
**Algorithm 1:** Multi-attribute decision-making.
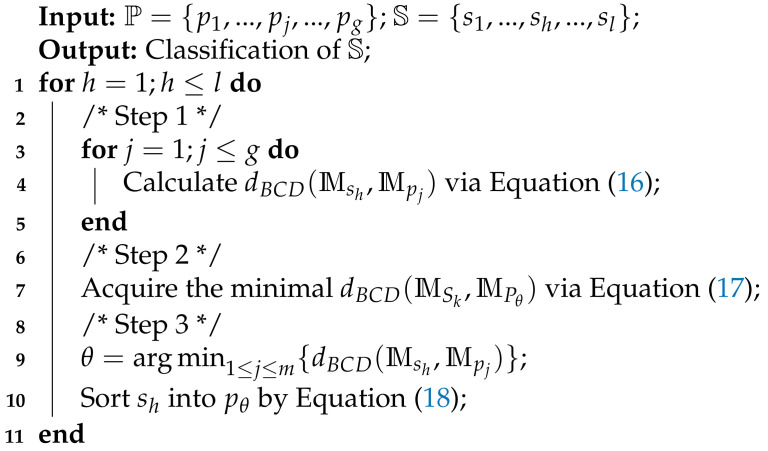


### 5.2. Application in Medical Diagnosis Under the Smart IoT Environment

Background: Consider a medical diagnosis decision-making problem under the smart IoT environment with three disease types, each of which has three attributes. Specifically, the given disease types $P=\{p_{1},p_{2},p_{3}\}$ and to be determined $s_{1}$ in S in terms of three attributes $\left\{x_{1},x_{2},x_{3}\right\}$ are modeled by the CBBAs from the sensor data under the smart IoT environment, shown in Table 3 and Table 4, respectively. Then, we need to figure out which disease $s_{1}$ is most likely to suffer from $\left\{p_{1},p_{2},p_{3}\right\}$.

The implementation of Algorithm 1 is illustrated as follows:

Step 1: The BCDs between $s_{1}$ and $p_{1}$, $s_{1}$ and $p_{2}$, and $s_{1}$ and $p_{3}$ are calculated:
  $$\begin{array}{c} d_{BCD}\left(M_{s_{1}},M_{p_{1}}\right)=0.2157,\\ d_{BCD}\left(M_{s_{1}},M_{p_{2}}\right)=0.2337,\\ d_{BCD}\left(M_{s_{1}},M_{p_{3}}\right)=0.1525. \end{array}$$
Step 2: The minimal BCD is $d_{BCD}\left(M_{s_{1}},M_{p_{3}}\right)$:
  $$\begin{array}{c} d_{BCD}\left(M_{s_{1}},M_{p_{3}}\right)=0.1525. \end{array}$$
Step 3: $s_{1}$ is determined to be the most likely to suffer from the disease type of $p_{3}$:
  $$\begin{array}{cc} \theta & =3;\\ s_{1} & \leftarrow p_{3}. \end{array}$$

**Figure 2:**
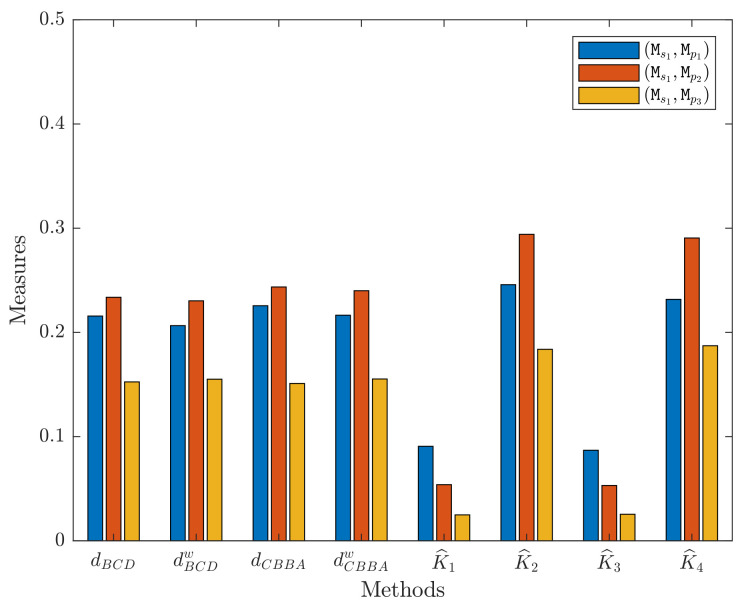
Comparison of different methods in the application.

The distance measure through the proposed method is shown in Table 5 and Figure 2, which has the ranking: $d_{BCD}\left(M_{s_{1}},M_{p_{3}}\right)<d_{BCD}\left(M_{s_{1}},M_{p_{1}}\right)<d_{BCD}\left(M_{s_{1}},M_{p_{2}}\right)$. Hence, sample $s_{1}$ is sorted into pattern $p_{3}$, which indicates that $s_{1}$ is most likely to suffer from the disease type of $p_{3}$.

### 5.3. Extension and Comparison

In Section 5.2, a medical diagnosis decision-making algorithm on the basis of the uniform scheme of the BCD is studied. In this section, taking into account that various attributes may have different weights in accordance with specific applications, the basis algorithm is extended, denoted as $d_{BCD}^{w}$:

(19) $$\begin{array}{cc} d_{BCD}^{w}\left(M_{S_{k}},M_{P_{j}}\right)\; & =\sum_{i=1}^{n}w_{i}d_{BCD}\left(M_{s_{h}}^{a_{\kappa }},M_{p_{j}}^{a_{\kappa }}\right)\\ \; & =\sum_{i=1}^{n}w_{i}\max_{A_{i}\subseteq \Phi }\left\{\left|BetC_{M_{s_{h}}^{a_{\kappa }}}\left(A_{i}\right)-BetC_{M_{p_{j}}^{a_{\kappa }}}\left(A_{i}\right)\right|\right\}, \end{array}$$

where $\sum_{i=1}^{n}w_{i}=1$.

It is worth noting that in Equation (19), $w_{i}$ can be determined according to specific applications, such as subjective weights provided by experts and objective weights calculated based on data-driven methods.

In order to validate the effectiveness of the proposed methods $d_{BCD}$ and $d_{BCD}^{w}$, both of them are compared with $d_{CBBA}$ and $d_{CBBA}^{w}$ of Xiao’s method [28,29] and ${\hat{K}}_{1}=1-K_{1}$, ${\hat{K}}_{2}=1-K_{2}$, ${\hat{K}}_{3}=1-K_{3}$, and ${\hat{K}}_{4}=1-K_{4}$ of Garg and Rani’s method [30]. In this application, the weight $w_{i}$ is set as [0.3,0.35,0.35,0.35] according to [30]. By implementing $d_{BCD}$, $d_{BCD}^{w}$, $d_{CBBA}$, $d_{CBBA}^{w}$, ${\hat{K}}_{1}$, ${\hat{K}}_{2}$, ${\hat{K}}_{3}$, and ${\hat{K}}_{4}$, the results are described in Table 5 and Table 6 and Figure 2.

To be specific, for $d_{BCD}^{w}$, Table 5 and Table 6 show that $d_{BCD}^{w}\left(M_{S},M_{P_{1}}\right)=0.2065$, $d_{BCD}^{w}\left(M_{S},M_{P_{2}}\right)=0.2303$, and $d_{BCD}^{w}\left(M_{S},M_{P_{3}}\right)=0.1551$, such that $d_{BCD}^{w}\left(M_{S},M_{P_{3}}\right)<d_{BCD}^{w}\left(M_{S},M_{P_{1}}\right)<d_{BCD}^{w}\left(M_{S},M_{P_{2}}\right)$. This result also means that $s_{1}$ is sorted into pattern $p_{3}$, and it is most likely to suffer from the disease type of $p_{3}$.

On the other hand, for $d_{CBBA}$ and $d_{CBBA}^{w}$, it can be seen that $d_{CBBA}\left(M_{S},M_{P_{3}}\right)=0.1526<d_{CBBA}\left(M_{S},M_{P_{1}}\right)=0.2256<d_{CBBA}\left(M_{S},M_{P_{2}}\right)=0.2436$; $d_{CBBA}^{w}\left(M_{S},M_{P_{3}}\right)=0.1553<d_{CBBA}^{w}\left(M_{S},M_{P_{1}}\right)=0.2165<d_{CBBA}^{w}\left(M_{S},M_{P_{2}}\right)=0.2400$. Hence, it is learned that both of the methods $d_{CBBA}$ and $d_{CBBA}^{w}$ sort $s_{1}$ as pattern $p_{3}$. Besides, for ${\hat{K}}_{1}$, ${\hat{K}}_{2}$, ${\hat{K}}_{3}$, and ${\hat{K}}_{4}$, it is obvious that ${\hat{K}}_{1}\left(M_{S},M_{P_{3}}\right)=0.0249<{\hat{K}}_{1}\left(M_{S},M_{P_{2}}\right)=0.0539<{\hat{K}}_{1}\left(M_{S},M_{P_{1}}\right)=0.0907$; ${\hat{K}}_{2}\left(M_{S},M_{P_{3}}\right)=0.1838<{\hat{K}}_{2}\left(M_{S},M_{P_{1}}\right)=0.2458<{\hat{K}}_{2}\left(M_{S},M_{P_{2}}\right)=0.2941$; ${\hat{K}}_{3}\left(M_{S},M_{P_{3}}\right)=0.0255<{\hat{K}}_{3}\left(M_{S},M_{P_{2}}\right)=0.0531<{\hat{K}}_{3}\left(M_{S},M_{P_{1}}\right)=0.0869$; ${\hat{K}}_{4}\left(M_{S},M_{P_{3}}\right)=0.1872<{\hat{K}}_{4}\left(M_{S},M_{P_{1}}\right)=0.2317<{\hat{K}}_{4}\left(M_{S},M_{P_{2}}\right)=0.2906$. Although ${\hat{K}}_{2}$ has a different ranking in terms of $\left(M_{S},M_{P_{1}}\right)$, $\left(M_{S},M_{P_{2}}\right)$, and $\left(M_{S},M_{P_{3}}\right)$ with other methods, its minimal measure is also regarded as ${\hat{K}}_{2}\left(M_{S},M_{P_{3}}\right)$. Therefore, the methods of ${\hat{K}}_{1}$, ${\hat{K}}_{2}$, ${\hat{K}}_{3}$, and ${\hat{K}}_{4}$ also sort $s_{1}$ as pattern $p_{3}$.

In summary, the proposed $d_{BCD}$- and $d_{BCD}^{w}$-based medical diagnosis decision-making algorithms are as effective as Xiao’s [28,29] and Garg and Rani’s methods [30] to address the medical diagnosis decision-making problem.

On the other hand, in this section, it simply adopts the weight from [30]. As discussed before, the weight can be generated with regard to specific applications: experts’ subjective weights and objective weights calculated based on collected data. For instance, for a kind of disease, it may have multiple attributes, each of which may have different weights. In this scenario, this weighted scheme provides a better applicability.

## 6. Conclusions

In this paper, a generalized betting commitment-based distance (BCD) is proposed to measure the difference among CBBAs in the complex plane framework of CET. Additionally, the defined BCD is analyzed, which has the properties of the nonnegativity, nondegeneracy, symmetry, and triangle inequality. We then prove that the BCD meets the distance axioms to be a strict distance metric. After that, the superiority of BCD is demonstrated through a comparison with other well-known methods. Besides, a basis and its extensional BCD-based multi-attribute decision-making algorithms are designed and then adopted to address a medical diagnosis problem under the smart IoT environment to reveal their effectiveness.

In summary, this is the first work to study a betting commitment-based distance between CBBAs in CET. Furthermore, the BCD is a generalized model of the traditional distance among the betting commitments of BBAs. In particular, when CBBAs reduce to the classical BBAs, the BCD degenerates into Liu’s distance among the betting commitments of BBAs. Therefore, the BCD offers a promising way to measure differences among CBBAs in CET, as well as handling medical diagnosis problems under the smart IoT environment.

## Figures and Tables

**Table 1:** The variation in $A_{i}$.

*i*	$A_{i}$
1	$\left\{\phi_{1}\right\}$
2	$\left\{\phi_{1},\phi_{2}\right\}$
3	$\left\{\phi_{1},\phi_{2},\phi_{3}\right\}$
4	$\left\{\phi_{1},\phi_{2},\phi_{3},\phi_{4}\right\}$
5	$\left\{\phi_{1},\phi_{2},\phi_{3},\phi_{4},\phi_{5}\right\}$
6	$\left\{\phi_{1},\phi_{2},\phi_{3},\phi_{4},\phi_{5},\phi_{6}\right\}$
7	$\left\{\phi_{1},\phi_{2},\phi_{3},\phi_{4},\phi_{5},\phi_{6},\phi_{7}\right\}$
8	$\left\{\phi_{1},\phi_{2},\phi_{3},\phi_{4},\phi_{5},\phi_{6},\phi_{7},\phi_{8}\right\}$
9	$\left\{\phi_{1},\phi_{2},\phi_{3},\phi_{4},\phi_{5},\phi_{6},\phi_{7},\phi_{8},\phi_{9}\right\}$
10	$\left\{\phi_{1},\phi_{2},\phi_{3},\phi_{4},\phi_{5},\phi_{6},\phi_{7},\phi_{8},\phi_{9},\phi_{10}\right\}$
11	$\left\{\phi_{1},\phi_{2},\phi_{3},\phi_{4},\phi_{5},\phi_{6},\phi_{7},\phi_{8},\phi_{9},\phi_{10},\phi_{11}\right\}$
12	$\left\{\phi_{1},\phi_{2},\phi_{3},\phi_{4},\phi_{5},\phi_{6},\phi_{7},\phi_{8},\phi_{9},\phi_{10},\phi_{11},\phi_{12}\right\}$
13	$\left\{\phi_{1},\phi_{2},\phi_{3},\phi_{4},\phi_{5},\phi_{6},\phi_{7},\phi_{8},\phi_{9},\phi_{10},\phi_{11},\phi_{12},\phi_{13}\right\}$
14	$\left\{\phi_{1},\phi_{2},\phi_{3},\phi_{4},\phi_{5},\phi_{6},\phi_{7},\phi_{8},\phi_{9},\phi_{10},\phi_{11},\phi_{12},\phi_{13},\phi_{14}\right\}$
15	$\left\{\phi_{1},\phi_{2},\phi_{3},\phi_{4},\phi_{5},\phi_{6},\phi_{7},\phi_{8},\phi_{9},\phi_{10},\phi_{11},\phi_{12},\phi_{13},\phi_{14},\phi_{15}\right\}$
16	$\left\{\phi_{1},\phi_{2},\phi_{3},\phi_{4},\phi_{5},\phi_{6},\phi_{7},\phi_{8},\phi_{9},\phi_{10},\phi_{11},\phi_{12},\phi_{13},\phi_{14},\phi_{15},\phi_{16}\right\}$
17	$\left\{\phi_{1},\phi_{2},\phi_{3},\phi_{4},\phi_{5},\phi_{6},\phi_{7},\phi_{8},\phi_{9},\phi_{10},\phi_{11},\phi_{12},\phi_{13},\phi_{14},\phi_{15},\phi_{16},\phi_{17}\right\}$
18	$\left\{\phi_{1},\phi_{2},\phi_{3},\phi_{4},\phi_{5},\phi_{6},\phi_{7},\phi_{8},\phi_{9},\phi_{10},\phi_{11},\phi_{12},\phi_{13},\phi_{14},\phi_{15},\phi_{16},\phi_{17},\phi_{18}\right\}$
19	$\left\{\phi_{1},\phi_{2},\phi_{3},\phi_{4},\phi_{5},\phi_{6},\phi_{7},\phi_{8},\phi_{9},\phi_{10},\phi_{11},\phi_{12},\phi_{13},\phi_{14},\phi_{15},\phi_{16},\phi_{17},\phi_{18},\phi_{19}\right\}$
20	$\left\{\phi_{1},\phi_{2},\phi_{3},\phi_{4},\phi_{5},\phi_{6},\phi_{7},\phi_{8},\phi_{9},\phi_{10},\phi_{11},\phi_{12},\phi_{13},\phi_{14},\phi_{15},\phi_{16},\phi_{17},\phi_{18},\phi_{19},\phi_{20}\right\}$

**Table 2:** Comparison of |K|, $d_{CBBA}$, and $d_{BCD}$ in terms of the three cases of the complex basis belief assignments (CBBAs) in Example 8.

Methods	Conflict Measures		
	Case 1: $\left(M_{1}^{C_{1}},M_{2}^{C_{1}}\right)$	Case 2: $\left(M_{1}^{C_{2}},M_{2}^{C_{2}}\right)$	Case 3: $\left(M_{1}^{C_{3}},M_{2}^{C_{3}}\right)$
|K|	0.8400	0.2640	0.0000
$d_{CBBA}$	0.7071	0.5802	0.7242
$d_{BCD}$	0.7062	0.4380	0.6062

**Table 3:** The given patterns with multiple attributes modeled as CBBAs in the application.

P	X	CBBAs		
		M({y})	M({n})	M({y,n})
$p_{1}$	$a_{1}$	$0.9901e^{i\arctan(0.0101)}$	0	$0.0141e^{i\arctan(-1.0000)}$
	$a_{2}$	$0.8062e^{i\arctan(0.1250)}$	0	$0.2236e^{i\arctan(-0.5000)}$
	$a_{3}$	$0.7071e^{i\arctan(0.1429)}$	$0.1118e^{i\arctan(-0.5000)}$	$0.2062e^{i\arctan(-0.2500)}$
$p_{2}$	$a_{1}$	$0.9055e^{i\arctan(0.1111)}$	$0.1414e^{i\arctan(-1.0000)}$	0
	$a_{2}$	$0.9901e^{i\arctan(0.0101)}$	0	$0.0141e^{i\arctan(-1.0000)}$
	$a_{3}$	$0.9055e^{i\arctan(0.1111)}$	0	$0.1414e^{i\arctan(-1.0000)}$
$p_{3}$	$a_{1}$	$0.6083e^{i\arctan(0.1667)}$	$0.2062e^{i\arctan(-0.2500)}$	$0.2062e^{i\arctan(-0.2500)}$
	$a_{2}$	$0.8062e^{i\arctan(0.1250)}$	0	$0.2236e^{i\arctan(-0.5000)}$
	$a_{3}$	$0.9901e^{i\arctan(0.0101)}$	0	$0.0141e^{i\arctan(-1.0000)}$

**Table 4:** To be determined sample with multiple attributes modeled as CBBAs in the application.

S	X	CBBAs		
		M({y})	M({n})	M({y,n})
$s_{1}$	$a_{1}$	$0.5099e^{i\arctan(0.2000)}$	$0.3041e^{i\arctan(-0.1667)}$	$0.2062e^{i\arctan(-0.2500)}$
	$a_{2}$	$0.6083e^{i\arctan(0.1667)}$	$0.2062e^{i\arctan(-0.2500)}$	$0.2062e^{i\arctan(-0.2500)}$
	$a_{3}$	$0.8062e^{i\arctan(0.1250)}$	$0.1118e^{i\arctan(-0.5000)}$	$0.1118e^{i\arctan(-0.5000)}$

**Table 5:** The measures generated by different methods in the application.

Methods	Measures		
	$\left(M_{s_{1}},M_{p_{1}}\right)$	$\left(M_{s_{1}},M_{p_{2}}\right)$	$\left(M_{s_{1}},M_{p_{3}}\right)$
$d_{BCD}$	0.2157	0.2337	0.1525
$d_{BCD}^{w}$	0.2065	0.2303	0.1551
$d_{CBBA}$	0.2256	0.2436	0.1526
$d_{CBBA}^{w}$	0.2165	0.2400	0.1553
${\hat{K}}_{1}$	0.0907	0.0539	0.0249
${\hat{K}}_{2}$	0.2458	0.2941	0.1838
${\hat{K}}_{3}$	0.0869	0.0531	0.0255
${\hat{K}}_{4}$	0.2317	0.2906	0.1872

**Table 6:** The ranking and sorting obtained through different methods in the application.

Methods	Rankings	Sort
$d_{BCD}$	$d_{BCD}\left(M_{s_{1}},M_{p_{3}}\right)<d_{BCD}\left(M_{s_{1}},M_{p_{1}}\right)<d_{BCD}\left(M_{s_{1}},M_{p_{2}}\right)$	$p_{3}$
$d_{BCD}^{w}$	$d_{BCD}^{w}\left(M_{s_{1}},M_{p_{3}}\right)<d_{BCD}^{w}\left(M_{s_{1}},M_{p_{1}}\right)<d_{BCD}^{w}\left(M_{s_{1}},M_{p_{2}}\right)$	$p_{3}$
$d_{CBBA}$	$d_{CBBA}\left(M_{s_{1}},M_{p_{3}}\right)<d_{CBBA}\left(M_{s_{1}},M_{p_{1}}\right)<d_{CBBA}\left(M_{s_{1}},M_{p_{2}}\right)$	$p_{3}$
$d_{CBBA}^{w}$	$d_{CBBA}^{w}\left(M_{s_{1}},M_{p_{3}}\right)<d_{CBBA}^{w}\left(M_{s_{1}},M_{p_{1}}\right)<d_{CBBA}^{w}\left(M_{s_{1}},M_{p_{2}}\right)$	$p_{3}$
${\hat{K}}_{1}$	${\hat{K}}_{1}\left(M_{s_{1}},M_{p_{3}}\right)<{\hat{K}}_{1}\left(M_{s_{1}},M_{p_{2}}\right)<{\hat{K}}_{1}\left(M_{s_{1}},M_{p_{1}}\right)$	$p_{3}$
${\hat{K}}_{2}$	${\hat{K}}_{2}\left(M_{s_{1}},M_{p_{3}}\right)<{\hat{K}}_{2}\left(M_{s_{1}},M_{p_{1}}\right)<{\hat{K}}_{2}\left(M_{s_{1}},M_{p_{2}}\right)$	$p_{3}$
${\hat{K}}_{3}$	${\hat{K}}_{3}\left(M_{s_{1}},M_{p_{3}}\right)<{\hat{K}}_{3}\left(M_{s_{1}},M_{p_{2}}\right)<{\hat{K}}_{3}\left(M_{s_{1}},M_{p_{1}}\right)$	$p_{3}$
${\hat{K}}_{4}$	${\hat{K}}_{4}\left(M_{s_{1}},M_{p_{3}}\right)<{\hat{K}}_{4}\left(M_{s_{1}},M_{p_{1}}\right)<{\hat{K}}_{4}\left(M_{s_{1}},M_{p_{2}}\right)$	$p_{3}$

## References

[1] Qi J., Yang P., Newcombe L., Peng X., Yang Y., Zhao Z. (2020). An overview of data fusion techniques for Internet of Things enabled physical activity recognition and measure. Inf. Fusion.

[2] Huang K., Zhang Q., Zhou C., Xiong N., Qin Y. (2017). An efficient intrusion detection approach for visual sensor networks based on traffic pattern learning. IEEE Trans. Syst. Man Cybern. Syst..

[3] Alabdulkarim A., Al-Rodhaan M., Ma T., Tian Y. (2019). PPSDT: A novel privacy-preserving single decision tree algorithm for clinical decision-support systems using IoT devices. Sensors.

[4] Roy M., Chowdhury C., Aslam N. (2018). Designing transmission strategies for enhancing communications in medical IoT using Markov decision process. Sensors.

[5] Souza L.F.D.F., Silva I.C.L., Marques A.G., Silva F.H.D.S., Nunes V.X., Hassan M.M., Albuquerque V.H.C.D. (2020). Internet of Medical Things: An Effective and Fully Automatic IoT Approach Using Deep Learning and Fine-Tuning to Lung CT Segmentation. Sensors.

[6] Celesti A., Ruggeri A., Fazio M., Galletta A., Villari M., Romano A. (2020). Blockchain-Based Healthcare Workflow for Tele-Medical Laboratory in Federated Hospital IoT Clouds. Sensors.

[7] Takabayashi K., Tanaka H., Sakakibara K. (2019). Integrated Performance Evaluation of the Smart Body Area Networks Physical Layer for Future Medical and Healthcare IoT. Sensors.

[8] Mavrogiorgou A., Kiourtis A., Perakis K., Pitsios S., Kyriazis D. (2019). IoT in healthcare: Achieving interoperability of high-quality data acquired by IoT medical devices. Sensors.

[9] Depari A., Fernandes Carvalho D., Bellagente P., Ferrari P., Sisinni E., Flammini A., Padovani A. (2019). An IoT based architecture for enhancing the effectiveness of prototype medical instruments applied to neurodegenerative disease diagnosis. Sensors.

[10] Dempster A.P. (1967). Upper and Lower Probabilities Induced by a Multivalued Mapping. Ann. Math. Stat..

[11] Shafer G. (1976). A Mathematical Theory of Evidence.

[12] Deng Y. (2020). Information volume of mass function. Int. J. Comput. Commun. Control.

[13] Xiao F. (2020). EFMCDM: Evidential fuzzy multicriteria decision making based on belief entropy. IEEE Trans. Fuzzy Syst..

[14] Zhou M., Liu X.B., Chen Y.W., Yang J.B. (2018). Evidential reasoning rule for MADM with both weights and reliabilities in group decision making. Knowl.-Based Syst..

[15] Pan L., Deng Y. (2020). Probability transform based on the ordered weighted averaging and entropy difference. Int. J. Comput. Commun. Control.

[16] Yager R.R. (2019). Generalized Dempster–Shafer Structures. IEEE Trans. Fuzzy Syst..

[17] Song Y., Zhu J., Lei L., Wang X. (2020). A Self-adaptive combination method for temporal evidence based on negotiation strategy. SCIENCE CHINA Inf. Sci..

[18] Deng X., Jiang W. (2019). Evaluating green supply chain management practices under fuzzy environment: A novel method based on D number theory. Int. J. Fuzzy Syst..

[19] Deng X., Jiang W. (2019). A total uncertainty measure for D numbers based on belief intervals. Int. J. Intell. Syst..

[20] Yang J.B., Xu D.L. (2013). Evidential reasoning rule for evidence combination. Artif. Intell..

[21] Fujita H., Ko Y.C. (2020). A heuristic representation learning based on evidential memberships: Case study of UCI-SPECTF. Int. J. Approx. Reason..

[22] Deng Y. (2020). Uncertainty measure in evidence theory. Sci. China Inf. Sci..

[23] Deng Y. (2021). Deng entropy measure of quantum entanglement. chinaXiv.

[24] Fan L., Deng Y. (2020). Determine the number of unknown targets in Open World based on Elbow method. IEEE Trans. Fuzzy Syst..

[25] Li Y.X., Pelusi D., Deng Y. (2020). Generate two dimensional belief function based on an improved similarity measure of trapezoidal fuzzy numbers. Comput. Appl. Math..

[26] Mao S., Han Y., Deng Y., Pelusi D. (2020). A hybrid DEMATEL-FRACTAL method of handling dependent evidences. Eng. Appl. Artif. Intell..

[27] Luo Z., Deng Y. (2020). A matrix method of basic belief assignment’s negation in Dempster–Shafer theory. IEEE Trans. Fuzzy Syst..

[28] Xiao F. (2020). Generalization of Dempster–Shafer theory: A complex mass function. Appl. Intell..

[29] Xiao F. (2020). Generalized belief function in complex evidence theory. J. Intell. Fuzzy Syst..

[30] Garg H., Rani D. (2019). A robust correlation coefficient measure of complex intuitionistic fuzzy sets and their applications in decision-making. Appl. Intell..

[31] Han D., Dezert J., Yang Y. (2016). Belief interval-based distance measures in the theory of belief functions. IEEE Trans. Syst. Man Cybern. Syst..

[32] Yang Y., Han D. (2016). A new distance-based total uncertainty measure in the theory of belief functions. Knowl.-Based Syst..

[33] Jousselme A.L., Grenier D., Bossé É. (2001). A new distance between two bodies of evidence. Inf. Fusion.

[34] Jousselme A.L., Maupin P. (2012). Distances in evidence theory: Comprehensive survey and generalizations. Int. J. Approx. Reason..

[35] Bouchard M., Jousselme A.L., Doré P.E. (2013). A proof for the positive definiteness of the Jaccard index matrix. Int. J. Approx. Reason..

[36] Jiang W., Huang C., Deng X. (2019). A new probability transformation method based on a correlation coefficient of belief functions. Int. J. Intell. Syst..

[37] Xiao F., Cao Z., Jolfaei A. (2020). A novel conflict measurement in decision making and its application in fault diagnosis. IEEE Trans. Fuzzy Syst..

[38] Pan L., Deng Y. (2020). An association coefficient of belief function and its application in target recognition system. Int. J. Intell. Syst..

[39] Liu W. (2006). Analyzing the degree of conflict among belief functions. Artif. Intell..

[40] Xiao F. (2020). CED: A distance for complex mass functions. IEEE Trans. Neural Netw. Learn. Syst..

[41] Huang J., Wu X., Huang W., Wu X., Wang S. (2020). Internet of things in health management systems: A review. Int. J. Commun. Syst..

[42] Hossain M.S., Muhammad G. (2016). Cloud-assisted industrial Internet of Things (IIoT)–enabled framework for health monitoring. Comput. Netw..

[43] Gómez J., Oviedo B., Zhuma E. (2016). Patient monitoring system based on Internet of Things. Procedia Comput. Sci..

[44] Abawajy J.H., Hassan M.M. (2017). Federated internet of things and cloud computing pervasive patient health monitoring system. IEEE Commun. Mag..

[45] He D., Zeadally S. (2014). An analysis of RFID authentication schemes for internet of things in healthcare environment using elliptic curve cryptography. IEEE Internet Things J..

[46] Dimitrov D.V. (2016). Medical internet of things and big data in healthcare. Healthc. Inform. Res..

[47] Lomotey R.K., Pry J., Sriramoju S. (2017). Wearable IoT data stream traceability in a distributed health information system. Pervasive Mob. Comput..

[48] Zhang W., Yang J., Su H., Kumar M., Mao Y. (2018). Medical data fusion algorithm based on Internet of Things. Pers. Ubiquitous Comput..

[49] Dautov R., Distefano S., Buyya R. (2019). Hierarchical data fusion for Smart Healthcare. J. Big Data.

[50] Xiao F. (2020). Evidence combination based on prospect theory for multi-sensor data fusion. ISA Trans..

[51] Meng D., Liu M., Yang S., Zhang H., Ding R. (2018). A fluid–structure analysis approach and its application in the uncertainty-based multidisciplinary design and optimization for blades. Adv. Mech. Eng..

[52] Liu Z., Li G., Mercier G., He Y., Pan Q. (2017). Change detection in heterogenous remote sensing images via homogeneous pixel transformation. IEEE Trans. Image Process..

[53] Yager R.R. (2018). On Using the Shapley Value to Approximate the Choquet Integral in Cases of Uncertain Arguments. IEEE Trans. Fuzzy Syst..

[54] Gao X., Deng Y. (2020). The pseudo-pascal triangle of maximum Deng entropy. Int. J. Comput. Commun. Control.

[55] Li Y.F., Huang H.Z., Mi J., Peng W., Han X. (2019). Reliability analysis of multi-state systems with common cause failures based on Bayesian network and fuzzy probability. Ann. Oper. Res..

[56] Feng F., Cho J., Pedrycz W., Fujita H., Herawan T. (2016). Soft set based association rule mining. Knowl.-Based Syst..

[57] Witarsyah D., Fudzee M.F.M., Salamat M.A., Yanto I.T.R., Abawajy J. (2020). Soft Set Theory Based Decision Support System for Mining Electronic Government Dataset. Int. J. Data Warehous. Min. (IJDWM).

[58] Haruna K., Ismail M.A., Suyanto M., Gabralla L.A., Bichi A.B., Danjuma S., Kakudi H.A., Haruna M.S., Zerdoumi S., Abawajy J.H. (2019). A soft set approach for handling conflict situation on movie selection. IEEE Access.

[59] Yang J., Li S., Xu Z., Liu H., Yao W. (2020). An understandable way to extend the ordinary linear order on real numbers to a linear order on interval numbers. IEEE Trans. Fuzzy Syst..

[60] Fei L., Feng Y., Liu L. (2019). Evidence combination using OWA-based soft likelihood functions. Int. J. Intell. Syst..

[61] Jiang W., Cao Y., Deng X. (2020). A novel Z-network model based on Bayesian network and Z-number. IEEE Trans. Fuzzy Syst..

[62] Tian Y., Liu L., Mi X., Kang B. (2020). ZSLF: A new soft likelihood function based on Z-numbers and its application in expert decision system. IEEE Trans. Fuzzy Syst..

[63] Xiao F. (2020). On the maximum entropy negation of a complex-valued distribution. IEEE Trans. Fuzzy Syst..

[64] Xiao F. (2020). GIQ: A generalized intelligent quality-based approach for fusing multi-source information. IEEE Trans. Fuzzy Syst..

[65] Garg H., Rani D. (2019). Some results on information measures for complex intuitionistic fuzzy sets. Int. J. Intell. Syst..

[66] Lai J.W., Cheong K.H. (2020). Parrondo’s paradox from classical to quantum: A review. Nonlinear Dyn..

[67] Gao X., Deng Y. (2020). Quantum model of mass function. Int. J. Intell. Syst..

[68] Jiang W., Huang K., Geng J., Deng X. (2020). Multi-Scale Metric Learning for Few-Shot Learning. IEEE Trans. Circuits Syst. Video Technol..

[69] Deng J., Deng Y. (2021). Information volume of fuzzy membership function. Int. J. Comput. Commun. Control.

[70] Tang M., Liao H., Herrera-Viedma E., Chen C.P., Pedrycz W. (2020). A Dynamic Adaptive Subgroup-to-Subgroup Compatibility-Based Conflict Detection and Resolution Model for Multicriteria Large-Scale Group Decision Making. IEEE Trans. Cybern..

[71] Cao Z., Chuang C.H., King J.K., Lin C.T. (2019). Multi-channel EEG recordings during a sustained-attention driving task. Sci. Data.

[72] Liu P., Zhang X. (2020). A new hesitant fuzzy linguistic approach for multiple attribute decision making based on Dempster–Shafer evidence theory. Appl. Soft Comput..

[73] Liu Q., Tian Y., Kang B. (2019). Derive knowledge of Z-number from the perspective of Dempster–Shafer evidence theory. Eng. Appl. Artif. Intell..

[74] Xu X., Zheng J., Yang J.b., Xu D.l., Chen Y.w. (2017). Data classification using evidence reasoning rule. Knowl.-Based Syst..

[75] Pan Y., Zhang L., Wu X., Skibniewski M.J. (2020). Multi-classifier information fusion in risk analysis. Inf. Fusion.

[76] Fu C., Chang W., Yang S. (2020). Multiple criteria group decision making based on group satisfaction. Inf. Sci..

[77] Fei L., Lu J., Feng Y. (2020). An extended best-worst multi-criteria decision-making method by belief functions and its applications in hospital service evaluation. Comput. Ind. Eng..

[78] Kang B., Zhang P., Gao Z., Chhipi-Shrestha G., Hewage K., Sadiq R. (2020). Environmental assessment under uncertainty using Dempster–Shafer theory and Z-numbers. J. Ambient Intell. Humaniz. Comput..

[79] Liu Z., Pan Q., Dezert J., Han J.W., He Y. (2018). Classifier fusion with contextual reliability evaluation. IEEE Trans. Cybern..

[80] Xiao F. (2020). CEQD: A complex mass function to predict interference effects. IEEE Trans. Cybern..

[81] Xiao F. (2019). A distance measure for intuitionistic fuzzy sets and its application to pattern classification problems. IEEE Trans. Syst. Man Cybern. Syst..

[82] Fei L., Feng Y., Liu L. (2019). On Pythagorean fuzzy decision making using soft likelihood functions. Int. J. Intell. Syst..

[83] Xue Y., Deng Y., Garg H. (2020). Uncertain database retrieval with measure-based belief function attribute values under intuitionistic fuzzy set. Inf. Sci..

